# Interactive Association of Drugs Binding to Human Serum Albumin

**DOI:** 10.3390/ijms15033580

**Published:** 2014-02-27

**Authors:** Feng Yang, Yao Zhang, Hong Liang

**Affiliations:** State Key Laboratory Cultivation Base for the Chemistry and Molecular Engineering of Medicinal Resources, Ministry of Science and Technology of China, Guangxi Normal University, Guilin 541000, Guangxi, China; E-Mail: zhangyao1006@gmail.com

**Keywords:** human serum albumin, binding site, interactions of protein-ligands, interactive associations of drugs, structure of protein complex

## Abstract

Human serum albumin (HSA) is an abundant plasma protein, which attracts great interest in the pharmaceutical industry since it can bind a remarkable variety of drugs impacting their delivery and efficacy and ultimately altering the drug’s pharmacokinetic and pharmacodynamic properties. Additionally, HSA is widely used in clinical settings as a drug delivery system due to its potential for improving targeting while decreasing the side effects of drugs. It is thus of great importance from the viewpoint of pharmaceutical sciences to clarify the structure, function, and properties of HSA–drug complexes. This review will succinctly outline the properties of binding site of drugs in IIA subdomain within the structure of HSA. We will also give an overview on the binding characterization of interactive association of drugs to human serum albumin that may potentially lead to significant clinical applications.

## 1. Introduction

Human serum albumin (HSA), being the most abundant protein of blood plasma, has many important physiological functions. Among them, HSA regulates colloidal osmotic pressure, and transports numerous endogenous compounds such as fatty acids (FA), hormones, bile acids, amino acids, metals and toxic metabolites [1–6]. Additionally, there is a wide variety of drugs that are delivered to their targeting organs/tissues by binding with HSA [1,7–9]. Therefore, HSA not only protects the bound drugs against oxidation and influences the *in vivo* drug distribution, but also alters the pharmacokinetic and pharmacodynamic properties of drugs [7,10–13].

HSA is a single-chain, non-glycosylated polypeptide with a molecular weight of 66,500 Da containing 585 amino acids [1]. HSA is a helical protein with turns and extended loops, and resembles a heart shape, with approximate dimensions of 80 × 80 × 30 Å [1,14]. In general, drugs have two forms in circulation, namely, bound or unbound to plasma proteins. The unbound drugs can passively diffuse through the barriers constituted by endothelial cells into the organs where they are metabolized, biliary excretion or glomerular filtration in kidney [7,15,16]. The unbound drugs can also be distributed intracellularly via specific transport systems. Only free drug molecules interact with therapeutic targets to produce therapeutic effects [7,17]. In most instances, the unbound drug concentration of drug in the tissue depends on the unbound drug concentration in the plasma [7]. Thus, HSA–drug interactions are an important factor to understand the pharmacokinetics and pharmacological effects of drugs [18–22].

**Figure 1.**
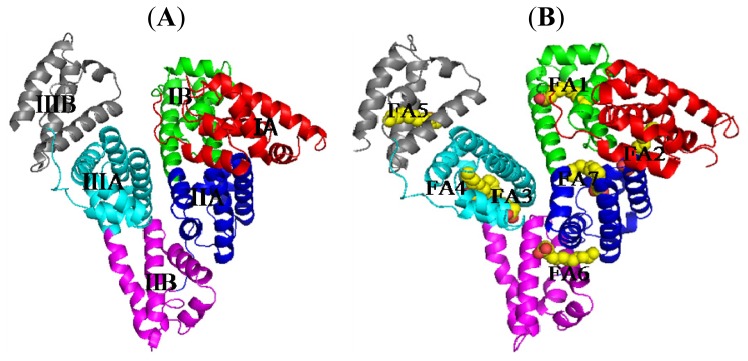
The overall structures of human serum albumin (HSA) (**A**) and HSA–fatty acids (FA) (**B**). Carbon atoms of FA molecule are shown in yellow; oxygen in red; nitrogen in blue; domain IA, in red; domain IB, in green; domain IIA, in blue; domain IIB, in magenta; domain IIIA, in cyan; domain IIIB, in grey.

The interactions between HSA and ligands have been extensively studied for several decades using a variety of methods [23–36]. However, since HSA is a flexible macromolecule featuring sophisticated binding sites for drugs, a detailed study of this system has been difficult to achieve. In fact, despite various HSA crystals were obtained several decades ago, the structure of this protein was difficult to elucidate due to the X-ray structure obtained from a crystal has low resolution [37–39]. It was not until 1992 that the HSA structure was really resolved by Carter *et al.* [40]. Furthermore, the structure of high resolution HSA complex was resolved in 1998 by curry *et al.* for the first time [41]. Subsequently, the structures of HSA forming complexes with other compounds have been popular and extensively studied by other groups, especially the group of Curry [42–66]. The structure of HSA reveals the presence of three domains, namely domains I (residues 1–195), II (196–383) and III (384–585), which, as predicted from amino acid sequence comparison, are not only topologically identical, but they also have similar three-dimensional structures [14,40]. The three domains are further divided into sub-domains A and B (Figure 1A). HSA contains 35 cysteine residues, and all of them except one, Cys34 (in domain I), are involved in disulfide bonds stabilizing the structure of HSA [14].

A fundamental characteristic of HSA is its surprising capacity to bind a large variety of drugs [1]. Taking into account the high concentration of HSA in plasma, the binding affinity of drugs to HSA is an important factor to be considered when designing and developing new drugs [67–71]. In addition, the interactive association of drugs that bind simultaneously to HSA can change the HSA binding behavior and potentially modulate the final therapeutic efficiency of the drugs [72]. Recent reported structures of HSA–ligands complexes not only clearly demonstrate the location of different drug binding sites on HSA, but they also revealed how several drugs interact with HSA [53,73–76]. Such knowledge on the binding properties of drugs to HSA is an important issue when analyzing the mechanisms affecting the pharmacological effects of these compounds. In this work, we focused on the nature of the HSA binding sites and the mechanisms of interactive association of drugs to HSA based on the available structural evidence found on HSA–drug complexes. Additionally, we highlight possible strategies that could be implemented to improve HSA-based delivery systems.

## 2. Binding Site of Drugs in IIA Subdomain of HSA

Since HSA has a limited number of high-affinity binding sites, detailed molecular information about these sites could be helpful in the assessment of cooperative effects during the binding of other drugs or endogenous ligands. Furthermore, the structural information of both high and low-affinity binding sites is useful when designing new drugs whether the aim is to avoid binding to HSA, or to make use of its depot function [67]. Therefore, the studies of drug binding sites on HSA have been important in the past and they keep being popular to date. Sudlow *et al.* did pioneering studies in this field by using a fluorescent probe displacement method in 1975 and 1976 [77,78]. With this method they showed through a screening, that there are two specific drug binding sites on HSA, namely, site I (also called the warfarin binding site) and site II (the benzodiazepine binding site). These excellent studies promoted the topology analysis and mapping of the drug binding sites on HSA. Using albumin fragments derived from pepsin and trypsin digestions, Bos *et al.* proposed that sites I and II are located in domains II and III, respectively [79,80]. Current crystallographic studies have proved that the majority of drugs bind to the above two main binding sites [53,74–76]. Certainly, these findings do not exclude the presence of other special binding sites on HSA [41,44,46,65,68]. However, in this review we only describe the main one binding site of HSA, namely, site I (IIA subdomain) owing to the following reasons. First of all, in the presence of fatty acids (FA), drugs bind preferentially to IB and IIA subdomain because IIIA subdomain is the strong binding site for fatty acids, thus fatty acids occupy this site inhibiting other drugs to bind here [81,82] (Figure 1B); In contrast, FA weakly bind to IIA subdomain, and is usually replaced by drugs. Additionally, interactive association of drugs binding to HSA usually occurs in IIA subdomain because site I is a big hydrophobic cavity that is possible to hold several drugs at the same time.

**Figure 2.**
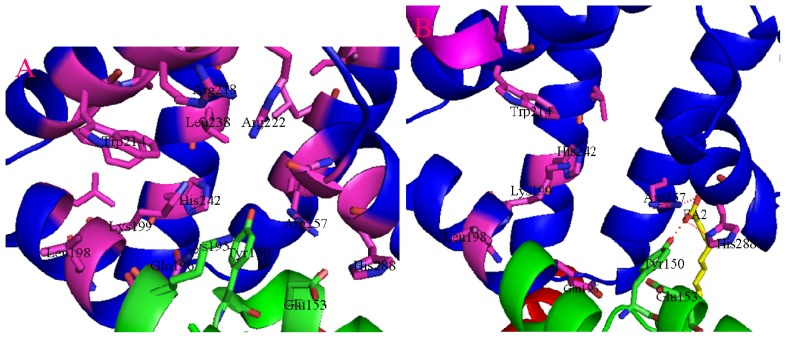
The binding environment of ligand site in IIA subdomain of HSA. (**A**) Site I; (**B**) Site I in HSA–FA. Domain IB, in green; domain IIA, in blue; Dashed line (red), hydrogen bond.

Site 1 is a preformed binding pocket within the core of subdomain IIA, which comprises six helices of the subdomain and a loop-helix feature (residues 148–154) contributed by subdomain IB. The interior of the pocket is hydrophobic, predominantly delimited by residues Trp214, Leu219, Phe223, Leu238, His242, Leu260, Ile264, Ser287, Ile290, and Ala291. However, it also contains also two clusters of polar residues, an inner cluster of residues toward the bottom of the pocket (Tyr150, His242, Arg257) and an outer cluster at the pocket entrance composed of Lys195, Lys199, Arg218, and Arg222 (Figure 2A). The large binding cavity is comprised of a central zone from which three distinct compartments extend. The distal end of the pocket is divided by residue Leu264 into left and right hydrophobic sub-chambers, whereas a third sub-chamber, delineated by residues Phe211, Trp214, Ala215, Leu238, as well as aliphatic portions of residues Lys199 and Arg218, protrudes from the front of the pocket (Figure 2A). Upon FA binding, residue Tyr150 from subdomain IB moves to interact with the carboxylate moiety of the lipid bound to a site that straddles domains I and II (fatty acid site FA2). This interaction helps to drive the relative rotation of domains I and II and has a large impact on only one side of drug site I. Extensive rearrangement of the H-bond network occurs, involving residues Tyr150, Glu153, Gln196, His242, Arg257, and His288, which opens a solvent channel between Tyr150 and Gln196, thus increasing the pocket volume and altering the distribution of polarity: the inner polar cluster is disrupted and partially neutralized by FA binding, whereas residue His242 is relatively unaffected (Figure 2B).

## 3. Interactive Association of Drug–Drug with HSA

**Figure 3.**
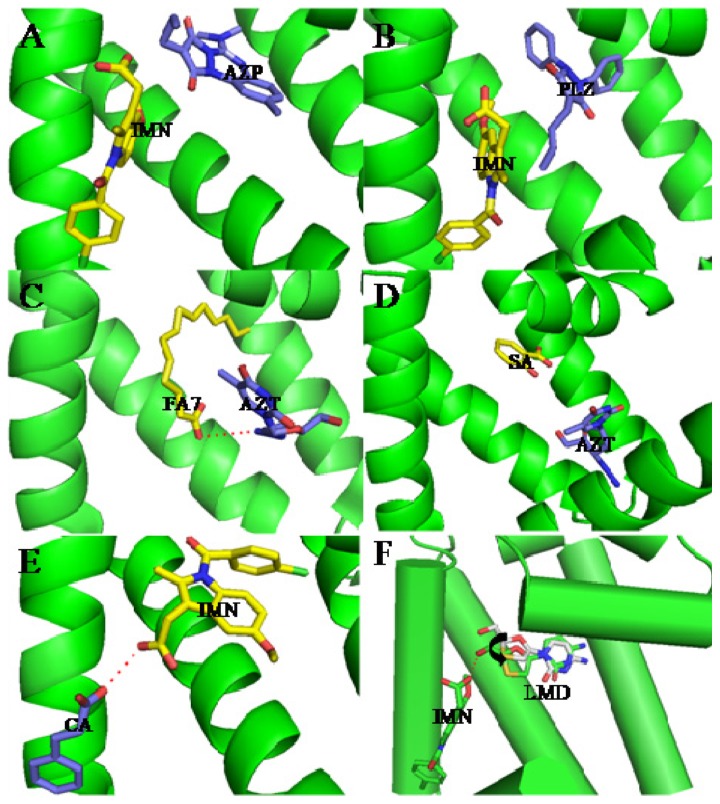
Structural basis of drug–drug co-binding to subdomain IIA of HSA. (**A**) azapropazone (AZP)–indomethacin (IMN); (**B**) phenylbutazone (PLZ)–IMN; (**C**) FA-3′-azido-3′-deoxythymidine (AZT); (**D**) salicylic acid (SA)–AZT; (**E**) IMN–cinnamic acid (CA); (**F**) Comparison of binding mode of lamivudine (LMD) to IIA subdomain of HSA in the presence/absence of IMN. Dashed line (red), hydrogen bond.

Drug–drug interactions at the protein-binding level can be useful for therapeutic purposes because alteration in protein binding may change the volume of distribution, clearance, and elimination of a drug and may modulate its therapeutic effect [8,72,83]. It was the first structures of drug–drug interactions with HSA resolved by Curry *et al.*, which provided a high value template for exploring drug–drug interactions [53]. Curry *et al.* initially suggested that indomethacin (IMN) would co-bind with some other site I specific compounds such as azapropazone (AZP), oxyphenbutazone, phenylbutazone (PLZ), 3,5-diiodosalicylic acid and tri-iodobenzoic acid by superposing the HSA–FA–IMN structure with those for other HSA–FA–drug complexes. To test this idea, PLZ–IMN and AZP–IMN double-drug were soaking into HSA–FA crystals. As a matter of fact, their experiment fit well with their hypothesis. The structures revealed that IMN and either AZP or PLZ do not displace one another from HSA, but simultaneously co-exist in the IIA subdomain (Figure 3A,B). Interestingly, the two drugs are slightly shifted if we compared them with their positions in the corresponding single drug complexes presumably as a result of drug–drug contacts [53]. The most striking effect of co-binding of these two drugs is the concerted rearrangement of Arg218 and Arg222, the principal effect of which is to substitute Arg222 instead of Arg218 as a binding partner for the carbonyl group of PLZ [53]. Additionally, Huang *et al.* think the presence of fatty acid is necessary for drugs’ co-binding in IIA subdomain due to fatty acid can induce conformational changes of HSA to create new sub-site [56]. For example, they observed that two compounds (3′-azido-3′-deoxythymidine (AZT) and FA) coexist in the IIA subdomain [56]. HSA–FA–AZT structure revealed that AZT does not displace FA7, but moves to a new subsite of the IIA subdomain which is different from the subsite of IMN (Figure 3A–C). FA7 is still at the centre of IIA subdomain, and forms a hydrogen bond with AZT (Figure 3C). This new AZT subsite is close to subdomain IB and is besieged by hydrophilic and polar amino acids, including Glu153, Ser192, Lys195, Gln196 from subdomain IB and Lys199, His242, Arg257, Glu292 from site I [56]. Interestingly, from the HSA–FA–AZT–salicylic acid complex structure, Huang *et al.* [56] also observed the coexistence of AZT with another drug (salicylic acid (SA)) in IIA subdomain. SA replaces FA7 and bind at the center of IIA subdomain; AZT still remains in its original position, but AZT has no contact with SA although AZT forms hydrogen bonds with Glu153 and Arg257 (Figure 3C,D). Therefore, Huang *et al.* suggest that site I of HSA can be divided into three non-overlapped subsites: a SA subsite, an IMN subsite and an AZT subsite [56]. Unfortunately, the above structures only showed that the binding mode and binding site of drugs can affect each other when they co-bind to the IIA subdomain, but how is their binding affinity? Namely, is the binding affinity of drugs increasing, decreasing or do not influence each other? Fluorescence quenching demonstrated that the binding affinity of IMN to HSA is stronger than that of cinnamic acid (CA). The structures of the corresponding HSA complexes revealed that IMN and CA have a common binding site in the IIA subdomain. What would happen if IMN and CA interact with HSA at the same time? The structure revealed that IMN is at the center of the binding site, which is different form binding properties of IMN in PLZ–IMN and AZP–IMN interactions, but CA reposition itself through an alternative binding in a new subsite (Figure 3A,B,E). However, both the binding modes and binding affinities of these drugs suffer changes. In the presence of IMN, the binding affinity of HSA for CA decreased about five times compared with the same measure in the absence of IMN (Table 1). On the other hand, the binding affinity of IMN to HSA in the presence of CA was enhanced 1.4 times compared to the same measure in the absence of CA. These differences are attributable to their corresponding interaction forces with HSA [74]. CA just forms one hydrogen bond with IMN, contacting fewer residues of HSA than CA without IMN. However, IMN not only forms hydrogen bonds and salt bonds with HSA, but also interacts with CA. Obviously, CA is helpful in the binding of IMN to the IIA subdomain because Gibbs free energy (Δ*G*) of IMN interacting with HSA–CA is less than that of IMN with HSA. Although IMN inhibits CA to bind IIA subdomain to some extent, IMN supports lamivudine (LMD) to bind with IIA subdomain in that Δ*G* of LMD directly binding to HSA is higher than that of LMD interacting with HSA–IMN (Table 1), which improves about two times the binding affinity of HSA for LMD compared to the same measure in the absence of IMN [64]. LMD rotates approximately 20° compared to LMD in HSA–FA–LMD structure, interacting with IMN (Figure 3F). To sum up, binding of a drug to HSA sometimes influences simultaneous binding of other drugs, thus their binding characterization may be affected by each other.

## 4. Interactive Association of Drug–Drug–Drug with HSA

**Figure 4.**
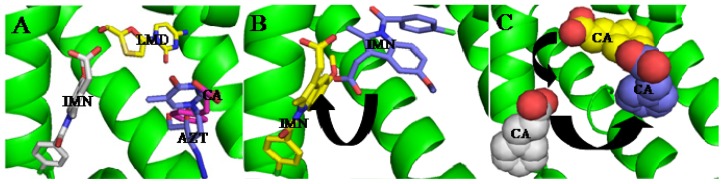
(**A**) Structural basis of IMN–CA–LMD co-binding to subdomain IIA of HAS; (**B**) the reposition route of IMN in the presence of CA and LMD; (**C**) the reposition route of CA in the presence of IMN and IMN–LMD. Dashed line (red), hydrogen bond.

In clinical practice, several drugs are often used to treat the disease synergistically, the binding strength of drugs to HSA plays a role in final therapeutic efficiency of the drugs [11]. Therefore, maybe we play optimal efficiency of drugs by interactions of drugs to regulate their binding affinity. The analysis of HSA structures has revealed that two drugs may co-bind the large hydrophobic cavity of the IIA subdomain (Figure 3). However, can several drugs co-bind this subdomain at the same time? To confirm the hypothesis, Yang *et al.* superposed the three structures corresponding to the complexes HSA–FA–IMN, HSA–FA–CA and HSA–FA–AZT, which showed that the three drugs have no overlap as they bind different positions of the IIA subdomain, which suggest that the three drugs could coexist simultaneously in the IIA subdomain [74]. As a matter of fact, the structure of the HSA–FA–CA–IMN–LMD complex revealed that the three drugs bind in the same binding site of the IIA subdomain [74]. Surprisingly, LMD does not bind to new subsite like AZT, but is at the center of IIA subdomain (Figure 4A). In fact, the binding affinity of CA and IMN to HSA are great stronger than that of LMD to HSA, but CA and IMN makes reposition (Figure 4B,C). The binding affinity of HSA for CA is a little affected by the presence or absence of IMN and LMD because CA moves to new subsite where AZT binds, forming hydrogen bonds with Glu153 and Ser192 (Figure 4A,C). However, the binding affinity of IMN to HSA is weaker than that of IMN in the presence of CA or CA and LMD. Obviously, the presence of the other drugs strengthens IMN to bind with IIA subdomain. Upon coexisting with other drugs, IMN is also easier to bind with HSA because the Gibbs free energy of IMN interacting with HSA is higher than that of IMN to HSA in the presence of other drugs.

**Figure 5.**
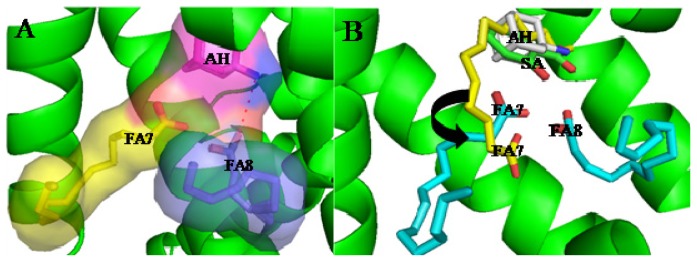
(**A**) Structural basis of AH and two FAs co-binding to subdomain IIA of HAS; (**B**) Binding mechanism of AH regulated by two FAs in IIA subdomain of HAS. Dashed line (red), hydrogen bond.

Treatment of brain disease is difficult to date because the majority of drugs cannot pass through the blood brain barrier, which hinders the drugs to reach their target and be clinically efficient [84–86]. Therefore, HSA delivery systems have been extensively studied for treating brain disease since HSA can pass through the blood brain barrier [87–92]. Disappointingly, HSA prefers to bind anionic but not cationic drugs in the IIA subdomain [93,94]. The structure of the complex HSA–lidocaine revealed that the drug binds to a unique site formed by residues from subdomain IB facing the central interdomain crevice [67]. The binding is mainly the result of cation–π interactions with Arg114, polar interaction with Lys190, and electrostatic attraction to Asp187 [67]. The IIA subdomain is a big hydrophobic binding site that can hold several drugs at the same time in theory. It raises the tempting possibility that we might be able to change the binding environment of the IIA subdomain to regulate the binding of cationic drugs which could be achieve by using other anionic drugs or by mutating nearby residues [73]. Yang *et al.* proved that these ideas are feasible. Fluorescence experiments demonstrated that a cationic drug, amantadine hydrochloride (AH), cannot bind the IIA subdomain when the molar ratio of FA to HSA is less than 1:7, but when the molar ratio of FA to HSA is at least 1:8 [73]. To explain the regulatory mechanism that describes the binding of AH to the IIA subdomain in presence of FA, the structure of the complex HSA–FA–AH was resolved (Figure 5A). The analysis of this structure revealed that one fatty acid (FA7) rotates about 90° and moves toward the bottom of binding cavity, which makes space to hold another fatty acid (FA8, Figure 5B). When both fatty acids are bound to the IIA subdomain, the binding of AH is stabilized by presence of interactions of the carboxyl group of FA8 that forms hydrogen bonds with the amino group of AH (Figure 5A). Interestingly, the binding position of AH overlaps the position where the drugs tend to bind (Figure 5B). Fortunately, the other anionic drugs also stabilize the binding of AH to the IIA subdomain [73]. Therefore, including modification to the IIA subdomain may be a promising strategy to enhance the drug binding and deliver capabilities of HSA especially when we want to redesign HSA to be carrier for drugs that cannot normally bind this protein [95]. These results provide evidence that it is possible to fully exploit the unique binding capacity of HSA’s IIA subdomain to achieve the simultaneous delivery of anionic and cationic drugs [73].

## 5. Conclusions

HSA is a non-toxic and non-antigenic endogenous protein that can carry different hydrophobic and hydrophilic drugs throughout the blood circulation system [95,96]. In addition, the drugs binding with HSA can directly form a “nano-drug” increasing drug bioavailability [2,95]. Thus, HSA-based delivery systems may be one of the most promising drug delivery systems [97–100]. To date, cancer treatment is still a big challenge for humans because anticancer drugs are associated with severe side effects and an inconvenient evolution of drug resistance during treatment [101–103]. Therefore, HSA-based delivery systems have been exploited to improve the targeting of anticancer drugs and decrease their side effects [104–107]. Additionally, we can conjugate HSA with active targeting molecules such as aptamers, antibodies, and various over-expressed receptors [108–110]. The ideal carrier should improve drugs efficiency and control the release rate of drugs at the right place [101]. The structural analysis of HSA complexes can guide us in the rational design of this protein by changing its drug binding ability using several strategies including, the modification of compounds’ structure, or the regulation of drugs to each other, or edit certain HSA amino acids rendering the HSA carrier more effective for drug delivery to the target site [96]. No doubt, HSA will be more extensively studied in various fields except that it will be more application in medicine field owing to its fascinating properties [95,111–119].

## Figures and Tables

**Table 1.** The binding constants (*K*) and free energy change (Δ*G*) of HSA and HSA complexes for drugs.

Drugs	Ref.	HSA/HSA complex	*K* (×10^4^ M^−1^)	Δ*G* (kJ/mol)
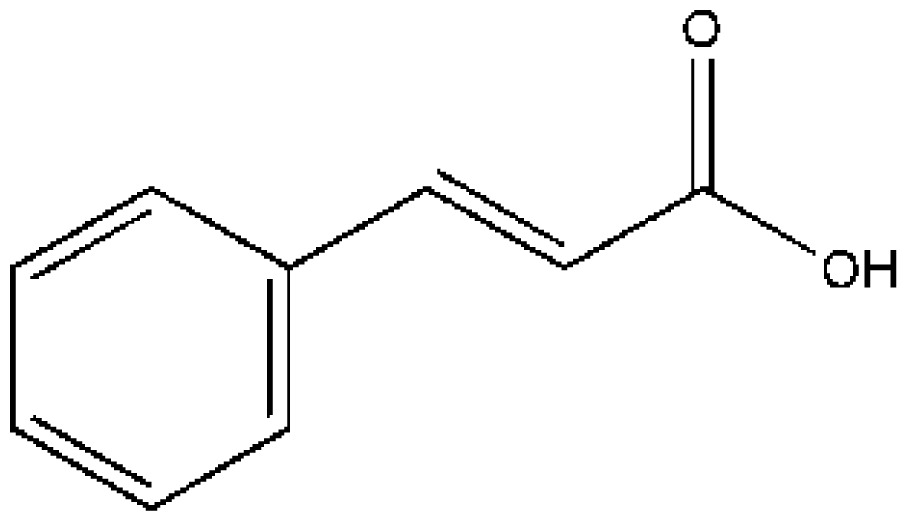 (CA)	[74]	HSA	5.329 ± 0.11	−26.965
		HSA–IMN	1.107 ± 0.06	−23.071
		HSA–IMN–LMD	3.296 ± 0.07	−25.774

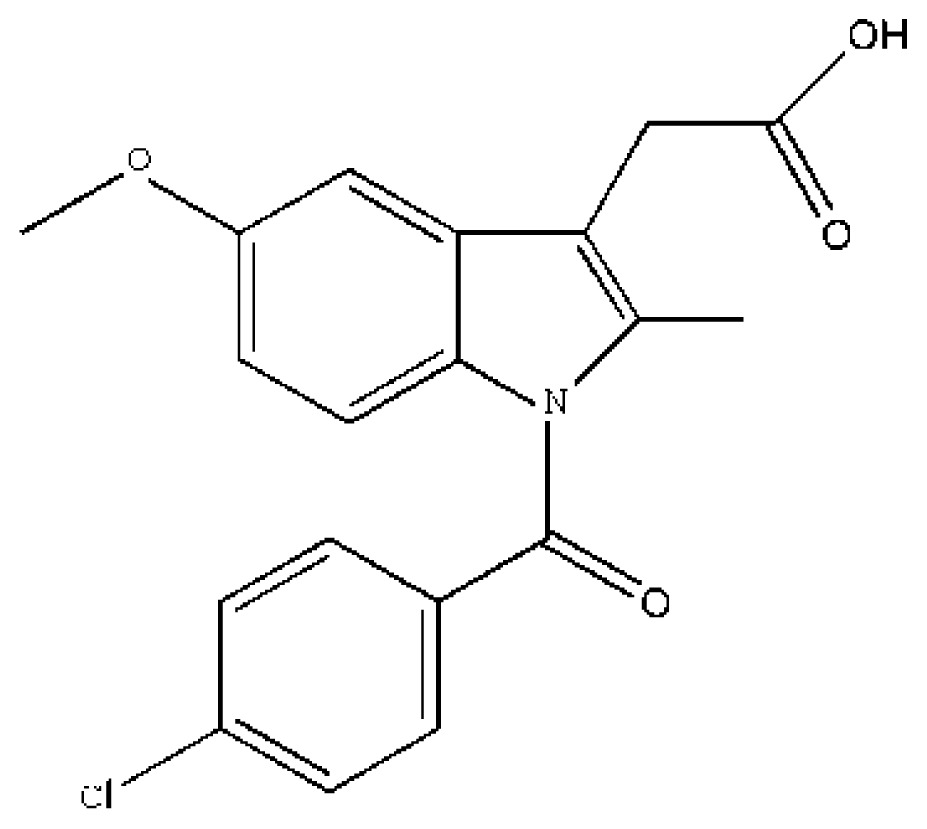 (IMN)	[74]	HSA	5.947 ± 0.09	−27.237
		HSA–CA	10.563 ± 0.13	−28.660
		HSA–CA–LMD	8.019 ± 0.11	−27.977

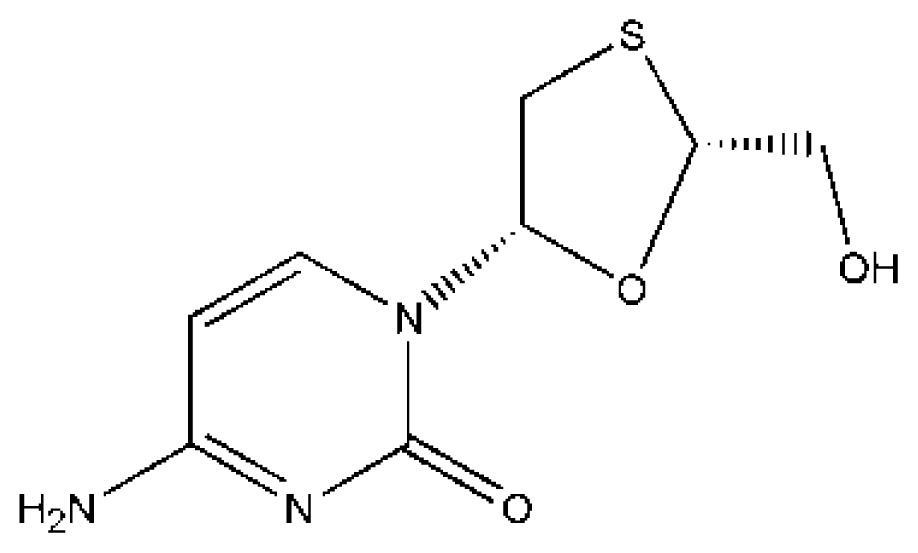 (LMD)	[64,74]	HSA	1.688 ± 0.16	−24.116
		HSA–IMN	4.07 ± 0.12	−27.85
		HSA–CA–IMN	3.220 ± 0.05	−25.717

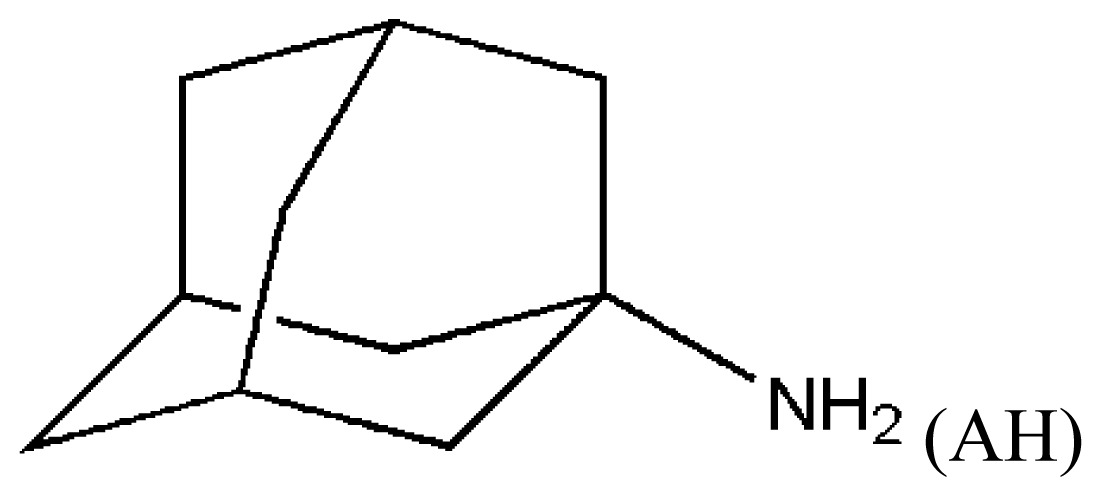 (AH)	[73]	HSA–myristate acids	1.909 ± 0.03	−24.421
		HSA–octanoic acid	2.745 ± 0.11	−25.321
